# Photothermal Performance of 2D Material-Based Nanoparticles for Biomedical Applications

**DOI:** 10.3390/nano15120942

**Published:** 2025-06-18

**Authors:** Amir Eghbali, Nikolay V. Pak, Aleksey V. Arsenin, Valentyn Volkov, Andrey A. Vyshnevyy

**Affiliations:** 1 Moscow Center for Advanced Studies, Kulakova str. 20, Moscow 123592, Russia; pak.nv@xpanceo.com; 2 Emerging Technologies Research Center, XPANCEO, Internet City, Emmay Tower, Dubai 00000, United Arab Emirates; arsenin@xpanceo.com (A.V.A.); vsv@xpanceo.com (V.V.)

**Keywords:** photothermal therapy, light-to-heat conversion, optical absorption, plasmonic resonance

## Abstract

Photothermal therapy (PTT) is one of the rapidly developing methods for cancer treatment based on the strong light-to-heat conversion by nanoparticles. Over the past decade, the palette of photonic materials has expanded drastically, and nanoparticle fabrication techniques can now preserve the optical response of a bulk material in produced nanoparticles. This progress potentially holds opportunities for the efficiency enhancement of PTT, which have not fully explored yet. Here we study the photothermal performance of spherical nanoparticles (SNs) composed of novel two-dimensional (2D) and conventional materials with existing or potential applications in photothermal therapy such as MoS_2_, PdSe_2_, Ti_3_C_2_, TaS_2_, and TiN. Using the Mie theory, we theoretically analyze the optical response of SNs across various radii of 5–100 nm in the near-infrared (NIR) region with a particular focus on the therapeutic NIR-II range (1000–1700 nm) and radii below 50 nm. Our calculations reveal distinct photothermal behaviors: Large (radius > 50 nm) nanoparticles made of van der Waals semiconductors and PdSe_2_ perform exceptionally well in the NIR-I range (750–950 nm) due to excitonic optical responses, while Ti_3_C_2_ nanoparticles achieve broad effectiveness across both NIR zones due to their dual dielectric/plasmonic properties. Small TiN SNs excel in the NIR-I zone due to the plasmonic response of TiN at shorter wavelengths. Notably, the van der Waals metal TaS_2_ emerges as the most promising photothermal transduction agent in the NIR-II region, particularly for smaller nanoparticles, due to its plasmonic resonance. Our insights lay a foundation for designing efficient photothermal transduction agents, with significant implications for cancer therapy and other biomedical applications.

## 1. Introduction

Photothermal therapy (PTT) is an emerging and minimally invasive therapeutic technique that utilizes photothermal transduction agents (PTAs) to convert light energy into heat for targeted tissue ablation [1,2]. This method has garnered significant interest for its potential applications in cancer treatment, where localized hyperthermia induced by PTAs can effectively destroy malignant cells while minimizing damage to the surrounding healthy tissues [3]. The efficiency of PTT depends heavily on the properties of PTAs—such as their geometry, size, composition, and biocompatibility—as well as on the characteristics of the illuminating light, including its wavelength [4]. While noble metals are widely used in PTT, alternative plasmonic materials like transition metal nitrides (TMNs) and van der Waals materials, such as transition metal dichalcogenides (TMDCs) and MXenes, whose atomic layers are weakly bound by van der Waals forces, offer several advantages, including lower toxicity [5], broad and tunable optical absorption [6], better photothermal stability [7], and cost-effectiveness [8].

Among nanoparticle morphologies, spherical nanoparticles (SNs) are particularly favored due to their ease of synthesis, enhanced cellular uptake, and lower cytotoxicity [9,10,11]. Additionally, nanoparticle size plays a crucial role in determining both the cellular uptake efficiency and the clearance rate from the bloodstream. Studies indicate that SNs with a radius of 10–50 nm offer optimal cellular uptake and prolonged circulation, thereby maximizing therapeutic efficacy [12,13,14], whereas larger nanoparticles are cleared faster by macrophages via the reticuloendothelial system. Moreover, SNs benefit from reduced aggregation. To diminish the immune response, nanoparticles are commonly covered with polyethylene glycol (PEG) [15]. Moreover, SNs benefit from reduced aggregation. At the same time, the main disadvantage of uniform SNs is the low tunability of the optical response which depends only on a single size parameter. Novel materials may help to overcome this drawback by providing an additional degree of freedom.

The selection of the operating wavelength is crucial for optimizing PTT performance. The near-infrared (NIR) region is particularly advantageous due to its deep tissue penetration and reduced scattering [16]. Within this spectrum, two therapeutic windows are recognized: the first, NIR-I, spans from 750 to 950 nm, while the second, NIR-II, lies slightly deeper into the near-infrared window from 1000 to 1700 nm [17]. NIR-II offers several benefits, including lower tissue absorption, reduced autofluorescence, and greater penetration depth [18,19]. These attributes make NIR-II an optimal spectral range for efficient photothermal heating, leading to improved therapeutic outcomes in biomedical applications. Over the last decade, considerable efforts have been spent to achieve stable production of highly crystalline SNs of van der Waals materials which preserve a strong excitonic optical response of the initial crystal [20,21,22,23]. Hence, the material palette available for nanoparticle production has significantly expanded, complicating the choice of suitable material for PTT. Novel materials feature exotic optical properties, such as record-high refractive indices and strong optical absorption in the vicinity of excitonic resonances [24,25,26]. Their potential for theranostic applications is not yet fully understood.

Beyond PTT, the enhancement of SN optical absorption is desirable for diagnostics. In particular, the efficiency of photoacoustic imaging (PAI), a hybrid imaging modality that combines the high contrast of optical imaging with the deep tissue penetration of ultrasound [27,28], strongly depends on the absorption in the near-infrared (NIR) region.

In this work, we conduct a detailed theoretical investigation of spherical nanoparticles for photothermal therapy. We identify the most effective materials, including novel van der Waals materials, for specific nanoparticle sizes (radius ≤ 100 nm) and operating wavelengths within the near-infrared (NIR) range. Special attention is given to the NIR-II region and particle radii ≤ 50 nm to optimize safety and efficacy.

## 2. Methods

### 2.1. Model for Temperature Rise

**Figure 1:**
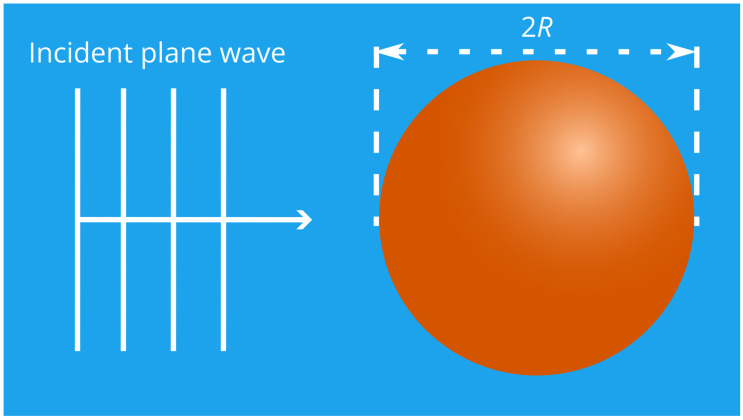
A plane wave incident upon a spherical nanoparticle of radius *R* embedded in a transparent medium.

At a steady state, where the temperature no longer changes with time in a solution containing SNs of radius *R*, the temperature increase (Δ*T*) due to nanoparticles is determined by the balance between energy absorption and heat dissipation in the surrounding medium. Mathematically, this relationship is given by

(1) $$\Delta T=\frac{Q_{0}f_{N}Vr}{NuAk}$$

where *Q*_0_ = *I*_0_*σ*_abs_ represents the energy absorbed by a single nanoparticle (Figure 1), where *σ*_abs_ is the absorption cross-section calculated using Mie theory [29] implemented in the self-written software. The incident radiation intensity *I*_0_ is set to 3.2 × 10^5^ W/m^2^, and the nanoparticle concentration is given by *f_N_* = 4.24 × 10^14^ (*R*_0_/*R*)^3^ m^−3^ based on the conditions used by Rastinehad et al. [30] for tumor therapy. We choose the reference radius as *R*_0_ = 50 nm. The solution’s geometry is characterized by its volume (*V*), surface area (*A*), and characteristic length (*r* = 1 cm). The thermal conductivity of water is *k* = 0.63 W/(m⋅K), and the Nusselt number is approximated as *Nu*∼5. Aiming to compare the optical performance of nanoparticles, we fixed the above parameters and scaled the nanoparticle concentration to be inversely proportional to the nanoparticle volume, thereby making the volume concentration constant. In practice, the concentration of nanoparticles in the target tumor will depend on the administered nanoparticle dose, the way they are introduced, the blood clearance rate, and the immune response. In the case when the practical conditions differ from the parameters listed here, our calculation results can be easily adjusted using Equation (1).

### 2.2. Absorption Cross-Section of Small Spheres

When the nanoparticle radius *R* is much smaller than the wavelength of incident light (*λ*/*R* ≫ 1), the electrodynamic effects of local-field enhancement become independent of the particle size. In this regime, retardation effects leading to radiative plasmon damping are negligible, and absorption is dominated by dipole interactions. The absorption cross-section can thus be expressed analytically using static dipole polarizability of a dielectric sphere, which is given by [31,32]

(2) $$\alpha =3\varepsilon_{0}V_{0}\frac{\varepsilon -\varepsilon_{h}}{\varepsilon +2\varepsilon_{h}}$$

Here, *V*_0_ is the volume of the particle, and *ε* represents the complex dielectric function of the particle material, while *ε_h_* is the dielectric constant of the host medium. The absorption cross-section is as follows:

(3) $$\sigma_{abs}=\frac{\frac{1}{2}Re\left(-i\omega \alpha E_{0}\cdot E^{*}\right)}{\frac{1}{2}Re\left(E_{0}H_{0}^{*}\right)}=V_{0}\frac{2\pi Im(\varepsilon )}{\lambda }\frac{9\varepsilon_{h}^{3/2}}{{\left(Re\left(\varepsilon \right)+2\varepsilon_{h}\right)}^{2}+Im{\left(\varepsilon \right)}^{2}}$$

where **E**_0_, **H**_0_, and **E** are the complex amplitudes of the electric and magnetic fields of the incident wave and the electric field inside the nanoparticle, respectively. Equation (3) indicates that the absorption cross-section is directly proportional to the volume of the particle, and it is related to the electrical conductivity *σ* of the particle material

(4) $$\sigma =\varepsilon_{0}\frac{2\pi c}{\lambda }Im\left(\varepsilon \right)$$

and field enhancement:

(5) $$\frac{{\left|E\right|}^{2}}{{\left|E_{0}\right|}^{2}}={\left|1-\frac{\alpha }{3\varepsilon_{0}V_{0}}\right|}^{2}=\frac{9\varepsilon_{h}^{2}}{{\left[Re\left(\varepsilon \right)+2\varepsilon_{h}\right]}^{2}+Im{\left(\varepsilon \right)}^{2}}$$

Rearranging Equation (3) yields a more geometric-friendly form:

(6) $${\left[Re(\varepsilon )\right]}^{2}+{\left[Im\left(\varepsilon \right)-b/\sigma_{abs}\right]}^{2}={\left(b/\sigma_{abs}\right)}^{2}$$

where *b* is

(7) $$b=9V_{0}\pi \varepsilon_{h}^{3/2}/\lambda .$$

Equation (6) reveals that, in the (Re(*ε*), Im(*ε*)) plane, the geometric locus of a constant absorption cross-section (*σ*_abs_) is a circle with a radius of *b*/*σ*_abs_ and a center at (−2*ε_h_*, *b*/*σ*_abs_). This geometric representation provides a powerful tool for visualizing absorption behavior, aiding in the identification of optimal material properties and wavelengths for small sizes. Note that even for small nanospheres, Equation (3) has limited applicability close to the localized plasmonic resonance at *ε* = −2*ε_h_*, where it formally yields an infinite absorption cross-section. In our work, Im(*ε*) is much larger than (2*πR*/*λ*)^3^, so Equation (3) is accurate.

## 3. Results and Discussion

We evaluate the photothermal response of spherical nanoparticles in water [33] (Figure 1) using five different nanoparticle materials—TaS_2_ [26], MoS_2_ [25], PdSe_2_ [34], Ti_3_C_2_ [35], and TiN [36]—selected for their distinct optical properties, making them promising for photothermal therapy.

### 3.1. Optical Heating Patterns

**Figure 2:**
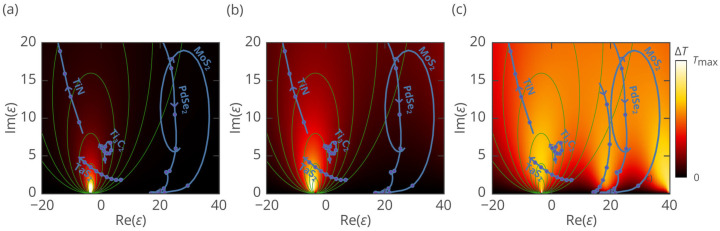
Calculated heating maps for spherical nanoparticles in water (refractive index: 1.327 [33]) with fixed wavelength (*λ*)-to-radius (*R*) ratios for different real and imaginary parts of permittivity: (**a**) *λ*/*R* = 100, (**b**) *λ*/*R* = 16, and (**c**) *λ*/*R* = 9. Directed blue lines depict the dispersion *ε*(*λ*) of different materials. Arrow directions correspond to the increase in wavelength from 650 nm to 1500 nm. Consecutive dots are 100 nm apart in wavelength. Thin green lines are lines of equal absorption within the static approximation plotted according to Equation (6). Note that Tmax differs for panels (**a**–**c**).

To put the performance of different materials into the same perspective, we calculated the temperature rise in nanoparticles having the same wavelength-to-radius ratios *λ*/*R* as a function of the complex refractive index, where *ε* = Re(*ε*) + *i*Im(*ε*). For details on the calculations see the Methods Section. The calculation results, presented in Figure 2, reveal the formation of hot zones related to resonant heating, a phenomenon where an incident wave efficiently couples to a nanoparticle eigenmode. This coupling leads to the enhanced local electric field, hence an increased optical absorption. Note that when the Im(*ε*) is smaller, the field enhancement is stronger due to the higher Q-factor of the corresponding resonance; however too small Im(*ε*) values will diminish optical absorption. Therefore, the maximum optical absorption is achieved at an optimal value of Im(*ε*) where radiative losses and absorption losses become equal [37].

Within our study range of particle radii from 5 to 100 nm and operating wavelengths from 650 to 1500 nm, ratios of *λ*/*R* = 9 and 100 correspond to large and small nanoparticles, respectively. Also, we added *λ*/*R* = 16 as a transitional case between these two extremes.

For *λ*/*R* = 100 (Figure 2a), a single electric dipole resonance with Re(*ε*) ≈ −3.5 is observed, which represents localized surface plasmon resonance. The absorption pattern for this ratio aligns well with the predictions of Equation (3).

For *λ*/*R* = 16 (Figure 2b), the same resonance is observed but shows slightly weaker agreement with Equation (3) due to the increased influence of radiative losses which are not accounted for by static approximation. Resonance is left-shifted on the Re(ε)–Im(ε) plane, leading to a slight red shift in the plasmonic resonance. Additionally, a trace of the magnetic dipole (MD) resonance is evident near the boundary of the simulation domain.

For *λ*/*R* = 9 (Figure 2c), the larger size of the structure relative to the wavelength leads to the occurrence of multiple resonances. When a material’s excitonic resonance in dielectric function is close to the Mie resonance hotspot, it results in Mie resonance-enhanced optical absorption. At Re(*ε*) ≈ −3.5, both electric dipole (ED) and electric quadrupole (EQ) plasmonic resonances are observed. MD resonance appears at Re(*ε*) ≈ 20, and at Re(*ε*) ≈ 40, both ED and magnetic quadrupole (MQ) resonances are present. The optimal Im(*ε*) values for dipole mode resonances are higher than those of *λ*/*R* = 16 and 100 due to increased radiative losses associated with larger relative sizes. Due to the strong coupling between material’s excitonic and geometric (Mie) resonances, the wavelength corresponding to the maximal optical absorption is red-shifted. The main reason for the red shift is the decrease in Im(ε) at which the maximal optical absorption is observed when the Mie resonance gets closer to the Re(ε) of the material. As a result, the optimal wavelength is shifted closer to the transparency band of highly refractive van der Waals materials capable of supporting Mie resonances.

To get a general sense of how various 2D materials perform across different sizes and wavelengths, we analyze the electric permittivity curves of the materials in Figure 2. The performance of small nanoparticles (Figure 2a) is dictated by how far the material properties are from plasmonic resonance. In this regard, MoS_2_ and PdSe_2_ nanoparticles demonstrate low optical absorption. In contrast, the dispersion lines of TaS_2_ (at *λ* ≈ 1300 nm), Ti_3_C_2_ (at *λ* ≈ 700, 1500 nm), and TiN (at *λ* ≈ 650 nm) pass close to the resonance, resulting in strong absorption.

As follows from Figure 2c, large nanoparticles are capable of Mie resonance-enhanced optical absorption. In particular, MoS_2_ nanoparticles support MD resonance (Re(*ε*) ≈ 20), followed by ED resonance (Re(*ε*) ≈ 40) at *λ* ≲ 700 nm. PdSe_2_ nanoparticle heating is due to MD resonance, which is particularly pronounced in the 800–1000 nm range. Meanwhile, the optical absorption of TaS_2_ (*λ* ≈ 1300 nm), Ti_3_C_2_ (*λ* ≈ 700, 1500 nm), and TiN (*λ* ≈ 650 nm) nanoparticles is again due to plasmonic resonances (Re(*ε*) ≈ −3.5).

### 3.2. Performance of 2D Materials for Photothermal Therapy and Imaging

**Figure 3:**
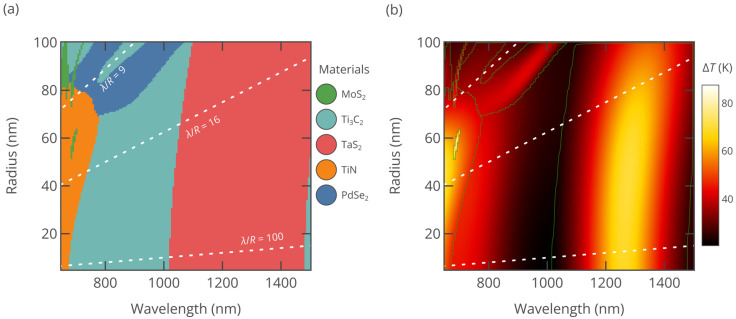
(**a**) Color map of the best-performing materials across radii ranging from 5 nm to 100 nm and wavelengths spanning 650 nm to 1500 nm. (**b**) Heating map for the selected materials. The green lines in panel (**b**) separate the areas of different materials. Dashed lines in panels (**a**,**b**) correspond to constant *λ*/*R* ratios in Figure 2.

**Figure 4:**
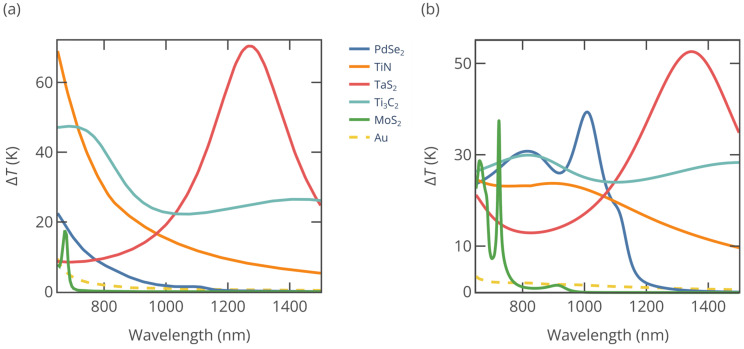
Heating as a function of wavelength for nanoparticles with radii of (**a**) *R* = 35 nm and (**b**) *R* = 100 nm composed of various materials. For comparison we added gold nanospheres of the same radii. The host medium is water.

To facilitate a systematic analysis and comparison of the 2D materials mentioned before—TaS_2_, MoS_2_, PdSe_2_, Ti_3_C_2_, and TiN—we divide the wavelength range into the NIR-I and NIR-II regions, while particle sizes are grouped as large (“L”: *R* > 50 nm) or small (“S”: *R* ≤ 50 nm). This classification creates four distinct zones on the radius–wavelength space: L-NIR-I, S-NIR-I, L-NIR-II, and S-NIR-II. In Figure 3 we plot the top-performing 2D materials and their temperature increase (Δ*T*) across these zones ($\sigma_{abs}$ and Δ*T* as a function of R and *λ* are included in Supplementary Table S1). For effective PTT, it is required that the tissue temperature reaches about 50 °C, which translates to a temperature rise ΔT of less than 20 °C [38]. As can be seen from Figure 3, the studied materials easily surpass this threshold. Meanwhile, Figure 4 demonstrates the heating performance of different materials for large (*R* = 100 nm) and small (*R* = 35 nm) nanoparticles within both the NIR-I and NIR-II regions. Below, we assess the performance of each 2D material based on the data presented in Figure 2, Figure 3 and Figure 4.

*MoS_2_ and other semiconducting TMDCs*: MoS_2_, a two-dimensional TMDC, exhibits pronounced excitonic effects due to its reduced dimensionality and strong Coulomb interactions [25,26]. Strong excitonic resonance manifests as the large loop in the Re(*ε*)–Im(*ε*) plane shows strong absorption and a high dielectric constant. As a result, with this material it is possible to achieve Mie resonance-assisted optical absorption, which requires the nanoparticle radius to be larger than 50 nm (Figure 3a). In this case, nanoparticles efficiently support ED, MD, and QD resonances. Figure 4 shows that with the increase in radius, resonance is red-shifted and becomes stronger, as expected from our previous analysis based on Figure 2. Other semiconducting TMDCs, e.g., WS_2_, WSe_2_, MoSe_2_, and MoTe_2_, show similar behavior [24,26], with excitonic resonances in NIR-I and no noticeable absorption in NIR-II, which is below their bandgap. Consequently, MoS_2_ and other TMDCs are best in narrow wavelength regions in the L-NIR-I zone. In PTT applications MoS_2_ nanoparticles may benefit from remarkable photothermal stability [20].

*PdSe_2_:* Similarly to semiconducting TMDCs, PdSe_2_ exhibits pronounced excitonic peaks, as reported in previous studies [25], with excitonic transitions forming a distinct arch in its dielectric function line in Figure 2. In the NIR-I window and the early NIR-II region, larger PdSe_2_ nanoparticles effectively sustain both MD and ED resonances. Although recent analysis of PdSe_2_ optical and electronic properties established that it is a semimetal [39], due to the momentum mismatch between semimetallic bands, its optical behavior in the near-infrared spectral range is closer to TMDC semiconductors rather than other van der Waals semimetals such as graphene. As a result, like other TMDC semiconductors, PdSe_2_ demonstrates strong performance in the L-NIR-I zone.

*TiN:* TiN is not a van der Waals material but has attracted great interest for decades due to its high melting point, chemical durability, and good conductivity, which makes it an “alternative plasmonic material” [40]. The TiN dielectric function crosses zero in the visible range, making it plasmonic in the NIR spectra region and hence attractive for PTT [41]. TiN SNs also show low cytotoxicity and high photothermal stability [41]. Furthermore, the dielectric function shifts away from the heating hotspots in Figure 2 as the wavelength increases. These characteristics explain why TiN exhibits its superior performance primarily in the S-NIR-I zone.

On a side note, many traditional plasmonic materials, such as noble metals and aluminum, undergo plasmonic transition deep in the visible range or even UV, so their uniform spherical nanoparticles perform quite badly as PTT agents. As seen from Figure 4, uniform gold nanoparticles yield temperature rise of less than 10 K throughout the whole NIR spectral region. To improve the performance, core–shell nanoparticles [30] or hollow gold shells [42], which require more sophisticated fabrication protocols [43], should be employed. The improvement in optical absorption comes at a price of increased size—specially designed gold nanoshell particles (Auroshell) for PTT have a diameter of about 150 nm [30].

*Ti_3_C_2_:* Ti_3_C_2_ belongs to MXenes, layered materials obtained by the etching of MAX phases [44], where M denotes the metal, A is an A-group atom, and X is carbon or nitrogen. As shown in Figure 2, the Ti_3_C_2_ dielectric function line features a loop associated with interband transitions [35], followed by an Re(*ε*) value of zero crossing near 1400 nm, signifying its metallic nature. As a result, the optical absorption of Ti_3_C_2_ nanoparticles in the near-infrared (NIR) region is due to proximity to localized plasmonic resonance. In the L-NIR-I area, they also benefit from the magnetic dipole (MD) response. The dual dielectric/plasmonic response makes Ti_3_C_2_ effective across all four zones: L-NIR-I, L-NIR-II, S-NIR-I, and S-NIR-II. Cytotoxicity studies of Ti_3_C_2_ MXene sheets show that they are more harmful to cancer cells than to normal ones [45]. Overall, the biocompatibility of Ti_3_C_2_ nanoparticles requires additional studies since the chemical activity and surface groups may change during nanoparticle synthesis.

*TaS_2_:* Unlike transition metal dichalcogenides composed of group IVB and group VIB transition metal atoms, TaS_2_ exhibits metallic behavior [46,47]. Tantalum is a biocompatible material; tantalum compounds are in medical use for more than 50 years [48]. Recent studies of PEG-TaS_2_ nanosheets demonstrated no significant cytotoxicity after 24–48 h of incubation [49]. As illustrated in Figure 2, Re(*ε*) for TaS_2_ transitions from positive to negative near 1100 nm, indicating a free-electron response [26]. Unlike Ti_3_C_2_, interband transitions in the TaS_2_ peak in the ultraviolet range do not influence the photoresponse in NIR. Furthermore, among the materials with Re(*ε*) < 0 whose dispersion curves were plotted in Figure 2, TaS_2_ demonstrates the lowest Im(*ε*), attributed to its uniquely low density of scattering states in the NIR, a result of its distinctive electronic band structure [47]. The combination of low Im(*ε*) and the dielectric function curve passing through Re(*ε*) = −2*ε*_h_ gives rise to an unparalleled plasmonic absorption peak in the NIR-II region, which is responsible for the remarkable performance of TaS_2_ nanoparticles in the L-NIR-II and S-NIR-II zones.

*Polydispersity:* The fabrication of nanoparticles by laser technologies, such as femtosecond laser ablation or laser fragmentation, results in a relatively broad distribution of their sizes. Although the size distribution can be narrowed by separating them via centrifugation, it is near impossible to obtain nanoparticles of the same radius. In this case, the temperature rise can be found by averaging the calculated temperature rise over the radius distribution function *df_N_* = *f*(*R*)*dR* as $\Delta T\left(\lambda \right)=\frac{1}{f_{N}\left(R_{0}\right)}\int_{0}^{\infty }\Delta T\left(\lambda ,\;R\right)R^{3}/R_{0}^{3}f\left(R\right)dR$. The averaging procedure smoothens the sharp features of Δ*T* (*λ*, *R*) such as the sharp Mie-excitonic resonances of large MoS_2_ nanoparticles somewhat diminishing their effectiveness. At the same time, broad plasmonic resonances of TiN, Ti_3_C_2_, and TaS_2_ remain unaffected by the polydispersity.

*Other applications:* SNs that strongly absorb light can have other applications beyond PTT. The same optical properties that make nanoparticles effective for PTT—such as their strong absorption in the NIR region—also make them well-suited for PAI, enabling simultaneous therapeutic and diagnostic applications.

### 3.3. In-Depth Study of the S-NIR-II Zone

To deepen our understanding of the physics underlying the S-NIR-II zone, we conduct a detailed investigation of TaS_2_—the most promising material among the examined 2D candidates. Our analysis unfolds in two stages: First, we compare the heating spectra obtained from both Equation (3) and Mie theory. Finally, we introduce a phenomenological model, drawing inspiration from Equations (3)–(6) and the underlying physics, to describe and explain the general behavior of the TaS_2_ nanoparticle in S-NIR-II.

**Figure 5:**
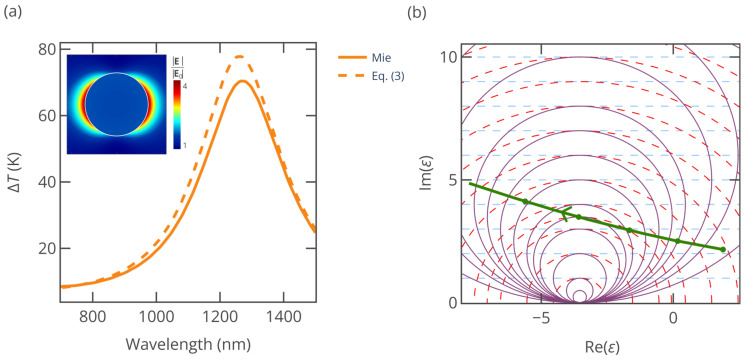
(**a**) Heating calculated using Mie theory for a nanoparticle with *R* = 35 nm (solid line) and Equation (3) (dashed line). The inset shows the electric field enhancement at *λ* = 1272 nm. (**b**) The directed blue-green line depicts the dispersion *ε*(*λ*) of TaS_2_ in the Re(*ε*)–Im(*ε*) plane. The arrow direction corresponds to the increase in *λ* from 1000 nm to 1500 nm. The solid purple lines are lines of equal absorption within the static approximation plotted according to Equation (6). The dashed blue lines are the lines of constant electrical conductivity (Equation (4)). The dashed red lines are the lines of constant electric field enhancement (Equation (5)).

The heating curve obtained from Equation (3) exhibits a peak temperature rise of ΔT = 78 K at a wavelength of *λ* = 1261 nm, while the exact curve calculated from the Mie theory shows a peak of Δ*T* = 70 K at *λ* = 1272 nm (Figure 5a). In the inset, we have the electric field enhancement at *λ* = 1272 nm. Given the small nanoparticle radius (*R* = 35 nm), the result from Equation (3) closely aligns with that of Mie theory. The observed blue shift in the peak position of Equation (3) relative to Mie theory, along with its higher temperature rise, can be attributed to the quasistatic approximation used in Equation (3), which neglects radiation losses and dynamic depolarization [50]. Indeed, according to the long-wavelength approximation [51], plasmonic resonance occurs at $\varepsilon /\varepsilon_{h}=-2-2.4\varepsilon_{h}{\left(2\pi R/\lambda \right)}^{2}$, which has a lower real part of dielectric permittivity; hence it is attained at a slightly higher wavelength.

As the wavelength increases, the TaS_2_ dispersion curve crosses the purple circles in Figure 5b with progressively smaller radii, which means a higher absorption cross-section. However, between 1200 nm and 1300 nm, this trend reverses, indicating that the absorption peak wavelength lies within this interval. A similar pattern emerges with the dashed red circles in Figure 5b, which indicate the field enhancement: as the wavelength increases, the TaS_2_ dispersion curve moves toward smaller radii but again reverses between 1200 nm and 1300 nm, thus locating the highest field enhancement resonance in this same wavelength range. An increase in Im(*ε*), represented by the dashed blue lines in Figure 5b, intensifies ohmic losses within the nanoparticle, reducing the internal-to-incident electric field ratio. As illustrated in the inset of Figure 5a, while a significant field enhancement of |**E**|/|**E**_0_| = 4 occurs around the nanoparticle surface at *λ* = 1272 nm, the internal field enhancement remains weak with |**E**|/|**E**_0_| = 1.6.

It should be noted that Figure 5b should not be used for the exact determination of the absorption peak, e.g., by finding the purple circle tangent to the dispersion line. According to Equations (6) and (7), the absorption associated with the circle is wavelength-dependent, so a larger circle can correspond to higher absorption if the wavelength is sufficiently smaller; thus the exact maximum is slightly blue-shifted from the tangent point. Nonetheless, it is useful for the illustration of the underlying physical mechanisms driving optical absorption in small nanoparticles.

The performance of small nanospheres is eventually determined by how small Im[*ε*(*ω*)] is when Re[*ε*(*ω*)] = −2*ε_h_*. It is instructive to roughly estimate the lower limit of Im(*ε*). In the visible and near-infrared range, negative permittivity is achieved thanks to free electron gas in metallic materials [52]. The dispersion law of these materials is usually described by $\varepsilon \left(\omega \right)=\;\varepsilon_{b}\left(\omega \right)-\omega_{p}^{2}/\left(\omega^{2}+i\Gamma \omega \right)$, where *ε_b_*(*ω*) is the contribution of bound electrons, and the second term is the free carrier response, with *ω_p_* and Γ being the plasma frequency and scattering rate. Plasmonic resonance is determined by the following condition: $-2\varepsilon_{h}=Re\left(\varepsilon_{b}\right)-\omega \omega_{p}^{2}/(\omega^{3}+\Gamma^{2}\omega )$. At the same time, $Im\left(\varepsilon \right)=Im\left(\varepsilon_{b}\right)+\Gamma \omega_{p}^{2}/\left(\omega^{3}+\Gamma^{2}\omega \right)$, from which we immediately obtain $Im\left(\varepsilon \right)=Im\left(\varepsilon_{b}\right)+\left[2\varepsilon_{h}+Re\left(\varepsilon_{b}\right)\right]\Gamma /\omega$. From this equation, it is easy to see conditions which must be met to achieve minimal Im(*ε*). First, no interband transition channels should be present to ensure Im(*ε_b_*) = 0. Second, Γ should be as small as possible. This value is determined by electron–phonon interactions and electron–electron scattering, and although they depend on details of electron–phonon and electron–electron coupling and the electronic density of states, a good estimate is Γ = 8 × 10^13^ s^−1^, which corresponds to the best-performing gold films [53]. Finally, one must achieve a minimal Re(*ε_b_*) value, ideally, close to one. The main difficulty is that the influence of optical transitions at the characteristic frequency *ω*_tr_ on Re(*ε*), according to the Kramers–Kronig relations [54], decays as 1/(*ω*_tr_ − *ω*) and may result in high Re(*ε_b_*) even when they are far from the interband absorption edge. A prominent illustration of this principle is MoS_2_ which has *ε* ≈ 17 in the near-infrared range. Assuming minimal possible Re(*ε_b_*) = 1 and Im(*ε_b_*) = 0, we obtain Im(*ε*) > 0.25 at a wavelength of 1300 nm, corresponding to the center of the NIR-II band. This value is an order of magnitude below that for TaS_2_, which gives a modest hope that even better materials for theranostic applications can be found in the future.

## 4. Conclusions

This study presents a comprehensive theoretical analysis of the photothermal response of spherical nanoparticles (SNs) composed of promising materials—TaS_2_, MoS_2_, PdSe_2_, Ti_3_C_2_, and TiN—across various sizes and near-infrared (NIR) wavelengths. These materials represent the families of TMDC semiconductors, MXenes, alternative plasmonic materials, and two-dimensional metals and semimetals. Our findings reveal distinct photothermal performance across different materials and spectral zones: MoS_2_ (and other TMDC semiconductors) excels in the L-NIR-I zone due to its exciton-driven effects, enabling strong magnetic dipole (MD), electric dipole (ED), and quadrupole (QD) resonances. PdSe_2_ also performs well in the L-NIR-I zone, driven by pronounced excitonic peaks and a metallic Drude response that enhances MD and ED resonances. TaS_2_ emerges as the top performer in the NIR-II region, particularly for smaller nanoparticles, due to its unique plasmonic absorption properties and minimal radiative losses. Ti_3_C_2_ demonstrates versatility across all spectral zones, supported by its dual dielectric/plasmonic response and interband transitions, while TiN shows superior performance in the S-NIR-I zone due to its plasmonic response at shorter wavelengths.

Among these materials, the van der Waals metal TaS_2_ stands out as the most promising candidate for photothermal therapy (PTT), achieving optimal photothermal heating efficiency in the NIR-II region. The theoretical framework developed in this study provides critical insights into the design of efficient photothermal transduction agents, underscoring the importance of material selection, nanoparticle size, and wavelength optimization. These findings advance the potential of PTT, particularly in cancer treatment, by enabling localized hyperthermia that selectively targets malignant cells while sparing healthy tissues. This research can be further developed in the direction of TaS_2_ and Ti_3_C_2_ nanoparticle fabrication and by testing their photothermal conversion, cytotoxicity, and biocompatibility, followed by in vivo PTT studies. Also, we predict the possibility of other materials which can outperform TaS_2_. This work paves the way for the development of next-generation photothermal transduction agents with enhanced therapeutic efficacy.

## Data Availability

The original contributions presented in the study are included in the article; further inquiries can be directed to the corresponding authors.

## Supplementary Materials

The following supporting information can be downloaded at https://www.mdpi.com/article/10.3390/nano15120942/s1, Table S1. Absorption cross-section *σ*_abs_ and temperature rise Δ*T* as a function of the wavelength and nanoparticle radius for different materials.
